# Supplementary material for: Dynamic palmitoylation events following T-cell receptor signaling
Source: Commun Biol. 2020 Jul 10;3:368. doi: 10.1038/s42003-020-1063-5 (PMC7351954; doi:10.1038/s42003-020-1063-5)
Supplement: Supplementary file 2 — Description of additional supplementary items [file 42003_2020_1063_MOESM2_ESM.pdf]

Description of Additional Supplementary Files

File Name: Supplementary Data 1
Description: Proteins enriched via ABE in unstimulated and/or anti-CD 3/anti-CD28 costimulated (10-min.) Jurkat T cells

File Name: **Supplementary Data 2**
Description: Raw data Figure 3

File Name: **Supplementary Data 3**
Description: Raw data Figure 2
