# Supplementary material for: Dynamic palmitoylation events following T-cell receptor signaling
Source: Commun Biol. 2020 Jul 10;3:368. doi: 10.1038/s42003-020-1063-5 (PMC7351954; doi:10.1038/s42003-020-1063-5)
Supplement: Supplementary file 6 — Reporting Summary [file 42003_2020_1063_MOESM6_ESM.pdf]

nature research

Corresponding author(s): Christian Freund

Last updated by author(s): Feb 20, 2020

Reporting Summary

Nature Research wishes to improve the reproducibility of the work that we publish. This form provides structure for consistency and transparency in reporting. For further information on Nature Research policies, see Authors & Referees and the Editorial Policy Checklist.

Statistics

For all statistical analyses, confirm that the following items are present in the figure legend, table legend, main text, or Methods section.

n/a	Confirmed	
☐	☒	The exact sample size (n) for each experimental group/condition, given as a discrete number and unit of measurement
☐	☒	A statement on whether measurements were taken from distinct samples or whether the same sample was measured repeatedly
☐	☒	The statistical test(s) used AND whether they are one- or two-sided<br>*Only common tests should be described solely by name; describe more complex techniques in the Methods section.*
☐	☒	A description of all covariates tested
☐	☒	A description of any assumptions or corrections, such as tests of normality and adjustment for multiple comparisons
☐	☒	A full description of the statistical parameters including central tendency (e.g. means) or other basic estimates (e.g. regression coefficient) AND variation (e.g. standard deviation) or associated estimates of uncertainty (e.g. confidence intervals)
☐	☒	For null hypothesis testing, the test statistic (e.g. F, t, r) with confidence intervals, effect sizes, degrees of freedom and P value noted<br>*Give P values as exact values whenever suitable.*
☒	☐	For Bayesian analysis, information on the choice of priors and Markov chain Monte Carlo settings
☒	☐	For hierarchical and complex designs, identification of the appropriate level for tests and full reporting of outcomes
☒	☐	Estimates of effect sizes (e.g. Cohen's d, Pearson's r), indicating how they were calculated

Our web collection on statistics for biologists contains articles on many of the points above.

Software and code

Policy information about availability of computer code

Data collection	Chromeleon LC Software, Xcalibur
Data analysis	MaxQuant (1.4.1.1), Python scripts

For manuscripts utilizing custom algorithms or software that are central to the research but not yet described in published literature, software must be made available to editors/reviewers. We strongly encourage code deposition in a community repository (e.g. GitHub). See the Nature Research guidelines for submitting code & software for further information.

Data

Policy information about availability of data

All manuscripts must include a data availability statement. This statement should provide the following information, where applicable:

- Accession codes, unique identifiers, or web links for publicly available datasets
- A list of figures that have associated raw data
- A description of any restrictions on data availability

Mass spectrometric raw files and data analysis scripts are available upon request.

Field-specific reporting

Please select the one below that is the best fit for your research. If you are not sure, read the appropriate sections before making your selection.

☒ Life sciences ☐ Behavioural & social sciences ☐ Ecological, evolutionary & environmental sciences

For a reference copy of the document with all sections, see nature.com/documents/nr-reporting-summary-flat.pdf

Life sciences study design

All studies must disclose on these points even when the disclosure is negative.

Sample size	For ABE quantification of palmitoylated proteins, a sample size of 4 biological replicates was chosen. For in vitro palmitoylation assays, at least 12 technical replicates were performed per protein construct.
Data exclusions	Known contaminants and E3 ligases were manually filtered out from the ABE enrichment data.
Replication	For palmitoylated proteins, multiple biological replicates were performed to ensure reproducible enrichment and quantification.
Randomization	Processing of ABE samples was performed randomized. For the OPPA assay plate positions were randomized
Blinding	Measurements of mass spectrometric samples were performed by a scientists who was not informed about the experimental setup.

Reporting for specific materials, systems and methods

We require information from authors about some types of materials, experimental systems and methods used in many studies. Here, indicate whether each material, system or method listed is relevant to your study. If you are not sure if a list item applies to your research, read the appropriate section before selecting a response.

Materials & experimental systems

n/a	Involved in the study	
☐	☒	Antibodies
☐	☒	Eukaryotic cell lines
☒	☐	Palaeontology
☒	☐	Animals and other organisms
☒	☐	Human research participants
☒	☐	Clinical data

Methods

n/a	Involved in the study	
☒	☐	ChIP-seq
☒	☐	Flow cytometry
☒	☐	MRI-based neuroimaging

Antibodies

Antibodies used	anti-CD3 IgM (C305) and anti-CD28 IgM hybridoma supernatants (Stefanie Kliche), 4G10 Platinum anti-phosphotyrosine (Millipore), anti-pERK (E-4) (Santa Cruz), anti-β-actin (AC-15) (SigmaAldrich), anti-LAT (Upstate (Millipore)) and anti-FLAG-HRP (Sigma Aldrich).The self-produced VAMP7 antibody was a kind gift of Dr. Andrew Peden (The University of Sheffield, UK). Anti-Flag(1/100) was from Sigma Aldrich (F3165). Anti-Giantin (1/100) was produced by the recombinant antibody platform of the Institut Curie, Paris, France. Anti–rabbit Ig Alexa Fluor 488 (1/200) and anti–mouse Ig Alexa Fluor 568 (1/200) antibodies were from Thermo Fisher Scientific (A11034 and A11004 respectively).
Validation	Antibodies were checked via Western blotting of appropriate test samples (e.g. Jurkat lysate).

Eukaryotic cell lines

Policy information about cell lines

Cell line source(s)	Jurkat E6.1 (Stefanie Kliche), self-generated stable Jurkat VAMP7 KO and lentiviral re-expression of VAMP7 WT and C183A
Authentication	Cell lines were authenticated for VAMP7 KO and re-expression by Western blotting and sequencing
Mycoplasma contamination	Cell lines were tested for mycoplasma contamination and were free from contamination
Commonly misidentified lines (See ICLAC register)	No commonly misidentified lines were used
